# The Extent of Honeycombing on Computed Tomography Cannot Predict the Treatment Outcome of Patients with Acute Exacerbations of Interstitial Lung Disease

**DOI:** 10.1155/2021/7456315

**Published:** 2021-11-16

**Authors:** Yurika Nishikawa, Yu Hara, Yoichi Tagami, Ryo Nagasawa, Kota Murohashi, Ayako Aoki, Katsushi Tanaka, Keisuke Watanabe, Nobuyuki Horita, Nobuaki Kobayashi, Masaki Yamamoto, Makoto Kudo, Takeshi Kaneko

**Affiliations:** ^1^Department of Pulmonology, Yokohama City University Graduate School of Medicine, Yokohama, Japan; ^2^Respiratory Disease Center, Yokohama City University Medical Center, Yokohama, Japan

## Abstract

**Background:**

The purpose of this retrospective study was to clarify whether the presence of honeycombing on computed tomography (CT) can affect the prognosis of patients with acute exacerbations (AEs) of interstitial lung diseases (ILDs).

**Methods:**

Clinical parameters including age, sex, Charlson Comorbidity Index Score (CCIS), blood biomarkers, and 3-month mortality were retrospectively compared between the CT honeycombing present and absent groups at the diagnosis of AEs of ILDs.

**Results:**

Ninety-five patients who were on corticosteroid pulse therapy were assessed. Though log-rank tests showed that Kaplan–Meier survival curves of the high and low ground-glass opacity (GGO) score groups differed significantly in 3-month mortality in patients with AEs of idiopathic ILDs (*P* = 0.007) and overall patients (*P* = 0.045), there was no significant difference between the CT honeycombing present and absent groups in patients with AEs of idiopathic ILDs (*P* = 0.472) and AEs of secondary ILDs (*P* = 0.905), as well as of overall patients (*P* = 0.600). In addition, whereas CCIS (OR, 1.436; 95% CI, 1.156–1.842; *P* < 0.001) was a significant predictor of 3-month mortality in the CT honeycombing absent group, serum LDH (OR, 1.005; 95% CI, 1.002–1.007; *P* = 0.001) was a significant predictor in the CT honeycombing present group.

**Conclusions:**

The clinical features of patients with or without honeycombing may differ due to the difference in prognostic factors, but these groups were found to have similar prognoses 3 months after AE onset, and clinicopathological examinations according to these groups are essential.

## 1. Introduction

An acute exacerbation (AE) of an interstitial lung disease (ILD) is a rapid deterioration of the respiratory condition that usually requires hospitalization and is associated with high in-hospital and postdischarge mortality [1]. It is widely known to occur in idiopathic pulmonary fibrosis (IPF), but it also occurs in other ILD patients such as non-IPF idiopathic interstitial pneumonias (IIPs) or connective tissue disease (CTD)-associated ILDs [2–6]. The typical pathological findings in patients with AEs of ILDs are known to be diffuse alveolar damage (DAD) superimposed on lung fibrosis (typically usual interstitial pneumonia (UIP)) with organizing pneumonia (OP), diffuse alveolar hemorrhage (DAH), pulmonary thromboembolism, lung cancer, and bronchopneumonia [7]. The high-resolution computed tomography (HRCT) findings of lung fibrosis in AE have been reported to range widely between typical honeycomb and reticular opacity without honeycomb [8, 9].

Honeycombing on HRCT is defined as clustered cystic airspaces of typically consistent diameter (3–10 mm, but occasionally larger) with thick and well-defined walls. It is usually accompanied by reticular fibrosis containing traction bronchiectasis [10]. Honeycombing has been described in diverse forms of ILDs such as chronic hypersensitivity pneumonitis, CTD-ILD, IPF, and unclassifiable/other ILDs [11]. Among ILD patients in the stable condition, the mean overall survival time has been reported to be shorter among those with CT honeycombing than among those without CT honeycombing [11]. However, there are few reports on the effect of the presence of CT honeycombing on the prognosis after the onset of AE [8]. The purpose of this retrospective study was to clarify whether the presence of CT honeycombing can affect the prognosis of patients with AEs of ILDs.

## 2. Materials and Methods

### 2.1. Study Location and Patients

This retrospective cohort study was performed at Yokohama City University Hospital and Yokohama City University Medical Center between 2014 and 2018. The medical data of 95 patients including acute or subacute IIPs, with AE of nonspecific interstitial pneumonia (NSIP) and IPF, cryptogenic organizing pneumonia (COP), or AE of CTD-ILDs treated with corticosteroid pulse therapy were assessed. Medical records at the time of diagnosis of AE were reviewed for data including age, sex, diagnosis of ILD, Charlson Comorbidity Index Score (CCIS), blood parameters such as partial pressure of oxygen in arterial blood/fraction of inspired oxygen (P/F ratio), lactate dehydrogenase (LDH; normal < 225 U/L), surfactant protein (SP)-D (normal < 110 ng/mL), Krebs von den Lungen (KL-6; normal < 500 U/mL), and treatment regimens, including anticoagulation therapy before steroid pulse therapy, steroid use before steroid pulse therapy, and macrolide therapy [12].

### 2.2. Diagnosis of ILDs

Subtypes of IIP were confirmed based on physical, serological, HRCT, and lung pathological findings, in accordance with the official statement for IIPs [13, 14]. Patients for whom lung biopsy could not be performed due to severe hypoxemia were diagnosed based on the HRCT classification [13, 14]. Typical HRCT patterns of IPF/UIP, NSIP, and COP were as follows: CT findings of IPF/UIP, subpleural, and basal predominant lung fibrosis including honeycomb, reticular opacity, and traction bronchiectasis with heterogenous distribution; NSIP, confluent bilateral lower lobe ground-glass opacities with marked traction bronchiectasis and lower lobe volume loss; COP, peripheral or bronchocentric consolidation and air bronchograms. The CTD-ILD diagnosis was confirmed by physical, serological, and HRCT findings consistent with ILD, and lung biopsy was undertaken to exclude other pulmonary diseases. Drug-induced ILD (DIILD) was diagnosed according to established criteria [15]. Also, we evaluated the chronic process of DIILD patients based on clinical information and HRCT findings. AE of ILD was defined as worsening of hypoxemia reflecting severely impaired gas exchange; worsening of dyspnea; newly appeared alveolar infiltration on radiography; and absence of alternative etiologies including pneumothorax, pulmonary embolism, infection, or heart failure [3–6].

### 2.3. HRCT Scorings

HRCT was performed at the time of diagnosis of AE. HRCT findings including ground-glass opacity (GGO) and honeycombing were assessed independently by two pulmonologists (experience-year: more than 10 years) and two radiologists (years of experience-year: more than 10 years).The findings of HRCT were evaluated using the semiquantitative scoring method described by Ooi et al. [16]. Abnormalities on HRCT images of the lungs were categorized as ground-glass opacity and consolidation, reticular fibrosis including traction bronchiectasis and honeycomb lung, and scored based on the ratios (%) of disease in each of the six lung lobes (0%: 0 point, 1–25%: 1 point, 26–50%: 2 points, 51–75%: 3 points, and 76%: 4 points). Global scores were calculated by adding the scores for each anomaly in all lobes. A fibrosis score was calculated as the sum of the reticular fibrosis and honeycomb scores. If there was a difference in the evaluation of CT findings, the score was decided by discussion between researchers.

### 2.4. Statistical Analysis

Data were analyzed statistically using JMP12 (SAS Institute Inc., Cary, NC, USA) and are shown as medians with 25th–75th percentiles or numbers (%). Groups were compared using the Wilcoxon rank-sum test or Pearson's chi-squared test. Optimal parameter cutoff values were determined from receiver operator characteristic (ROC) curves. Survival curves were generated using the Kaplan–Meier method and compared using log-rank tests. Predictors of 3-month mortality were determined using multiple stepwise regression analysis. Values with *P* < 0.05 were considered significant.

### 2.5. Study Approval

This research was performed in accordance with the Declaration of Helsinki and approved by the Institutional Review Board at Yokohama City University Hospital (approval number B171100003). In this retrospective study, informed consent was obtained by disclosing the clinical study with the description of the optout process (https://www.yokohama-cu.ac.jp/amedrc/ethics/ethical/fuzoku_optout.html).

## 3. Results

### 3.1. Patient Characteristics

Table 1 provides the clinical characteristics of the 95 enrolled patients with AEs of ILDs, all of whom received corticosteroid pulse therapy. The diagnoses of the patients were AEs of idiopathic ILDs in 62 patients (65%) and AEs of secondary ILDs in 33 patients (35%). Idiopathic ILDs consisted of 21 IPF patients (22%) and 41 non-IPF patients (43%), including 29 NSIP patients and 12 COP patients. Secondary ILD patients consisted of 19 CTD-ILD patients (20%), 13 DIILD patients (14%), and 1 CHP patient (1%). HRCT pattern of DIILD included 4 AE of UIP, 3 AE of NSIP, and 4 OP pattern. Three-month mortality of all enrolled patients was 24%. In addition, there were no significant differences of incidence of systemic steroid therapy of presteroid and poststeroid pulse and anticoagulant treatment between macrolide and without macrolide.  Table 2 provides the comparisons of the HRCT scores or ratios at the time of diagnosis of AE between survivors and nonsurvivors within 3 months. All of these scores or ratios including GGO and consolidation, reticular fibrosis, honeycomb scores, and the ratio of fibrosis score/total score did not differ significantly between these groups.

### 3.2. The Clinical Impact of GGO and Total HRCT Scores

**Figure 1:**
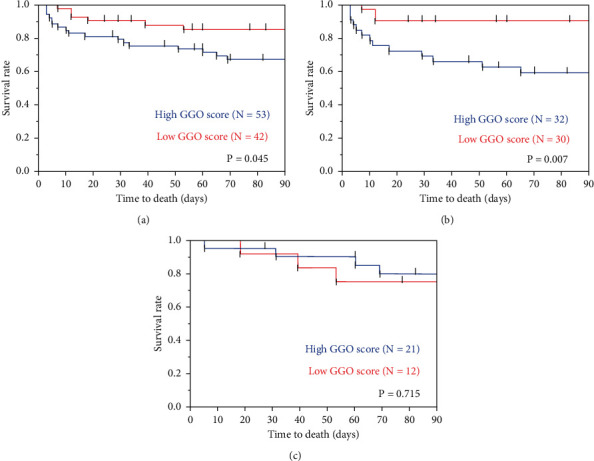
Comparison of patients with high and low GGO scores. The 95 patients assigned to groups with a low GGO (*N* = 42) or a high GGO (*N* = 53) score based on the optimal cutoff (10 points). Log-rank tests show that Kaplan–Meier survival curves of these groups differ significantly in patients with AE of idiopathic ILDs and overall patients, but not in those with AEs of secondary ILDs. AE, acute exacerbation; GGO, ground-glass opacity; ILD, interstitial lung disease. (a) Overall. (b) Idiopathic. (c) Secondary.

The area under the ROC curve (AUC) value was 0.600 in the evaluation of the GGO score as a predictor of 3-month mortality. Optimal parameter cutoff values determined from ROC analysis was 10 points (sensitivity is 74% and specificity 50% at this point). The 95 patients were assigned to groups with either low GGO (*N* = 42) or high GGO (*N* = 53) scores based on this cutoff (10 points). Log-rank tests showed that Kaplan–Meier survival curves of these groups differed significantly in patients with AEs of idiopathic ILDs (*P* = 0.007) and overall patients (*P* = 0.045), but not in those with AEs of secondary ILDs (Figure 1).

### 3.3. Clinical Impact of the Extent of Honeycombing on CT

**Figure 2:**
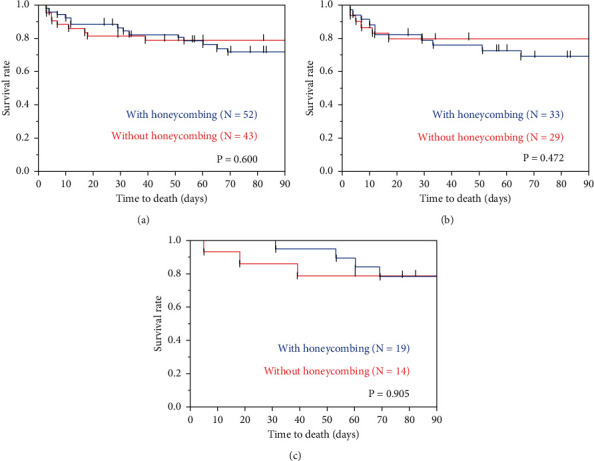
Comparison of patients with and without honeycombing. There is no significant difference in 3-month mortality between those with and without honeycombing in patients with AEs of idiopathic ILDs, AEs of secondary ILDs, and overall patients. AE, acute exacerbation; ILD, interstitial lung disease. (a) Overall. (b) Idiopathic. (c) Secondary.

**Figure 3:**
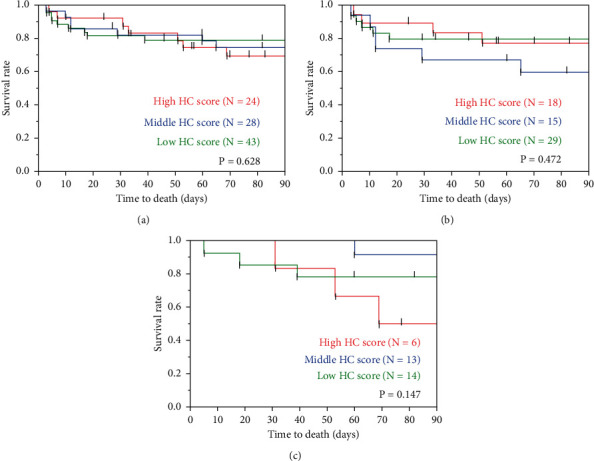
Comparison of patients with high, middle, and low honeycomb scores. In patients with AEs of idiopathic ILDs and secondary ILDs and overall patients, there is no significant difference in 3-month mortality among the groups with low (0 point), middle (1–4 points), and high (≥5 points) honeycomb scores. AE, acute exacerbation; ILD, interstitial lung disease. (a) Overall. (b) Idiopathic. (c) Secondary.

To evaluate the clinical impact of the extent of honeycombing on CT, clinical data including age, sex, diagnosis of ILD, CCIS, P/F ratio, LDH, SP-D, KL-6, and treatment regimens were compared between the patients with and without honeycombing on HRCT (Table 3). There were no significant differences between the two groups other than in the duration from ILD diagnosis to AE treatment, serum KL-6, and the frequency of use of macrolides. In addition, there was no significant difference in 3-month mortality between these two groups in patients with AEs of idiopathic ILDs (*P* = 0.600), AEs of secondary ILDs (*P* = 0.472), and overall patients (*P* = 0.905) (Figure 2). Also, in patients with AEs of idiopathic ILDs, AEs of secondary ILDs, and overall patients, there was no significant difference in 3-month mortality among the groups with low (0 point), middle (1–4 points), and high (≥5 points) honeycomb scores (Figure 3). Similar with the honeycomb score, there was no significant difference in 3-month mortality among the groups with low and high fibrosis scores including reticular fibrosis and honeycomb score (Figure S1).

### 3.4. Univariate and Multivariate Analyses for Evaluating Predictor of 3-Month Mortality

In both patients with and without honeycombing, clinical parameters including age, sex, CCIS, diagnosis of ILDs, P/F ratio, serum LDH and KL-6, and the GGO scores were evaluated. Whereas, CCIS (OR, 1.436; 95% CI, 1.156–1.842; *P* < 0.001) was a significant predictor of 3-month mortality in the CT honeycombing absent group, and serum LDH (OR, 1.005; 95% CI, 1.002–1.007; *P* = 0.001) was significant predictors in the CT honeycombing present group (Table 4).

## 4. Discussion

Honeycombing on HRCT is defined as clustered cystic airspaces of typically consistent diameter (3–10 mm, but occasionally larger) with thick and well-defined walls [10]. In the observational cohort study of data from America, the prevalence of CT honeycombing was reported to be about 30–40% in patients with CTD-ILD, IPF, chronic hypersensitivity pneumonia (CHP), and unclassified ILD, and CT honeycombing was associated with increased long-term mortality rate compared with no honeycombing [11]. Similar to these stable ILD patients, CT honeycombing is expected to be an important prognostic predictor in ILD patients having an AE, but there are few reports examining the relationship between CT findings including honeycombing, traction bronchiectasis, GGO, and consolidation [17]. Actually, Kishaba et al. demonstrated that, in patients with AEs of IPF, the GGO and consolidation score calculated from HRCT could be a significant predictor of 3-month mortality, but the honeycomb and traction bronchiectasis score could not [17]. The purpose of this retrospective study was to clarify whether the presence of CT honeycombing affects the prognosis of patients with AEs of ILDs.

It is not clear whether the presence of CT honeycombing affects the prognosis at the onset of AE [8]. In the present retrospective study that included 107 patients with AE-IPF, Kaplan–Meier analysis did not show any difference in overall survival between the UIP group and the possible UIP group, but the 30-day cumulative survival proportion was significantly higher in the UIP group than in the P-UIP group [8]. In the present study, there was no significant difference in 3-month mortality between with and without CT honeycombing groups among patients with AEs of idiopathic ILDs, AEs of secondary ILDs, and overall patients. This tendency was similar 6 months after AE onset (Figure S2). In addition, CCIS proved to be a significant prognostic factor in the former group, and serum LDH was significant in the latter group. Serum LDH has been previously mentioned as a prognostic factor in patients with AEs of IPF [17, 18]. However, the present study showed that the groups with and without CT honeycombing had different prognostic factors. Therefore, these suggest that the mechanism of disease progression in AE patients with and without CT honeycombing may be different, and it may be necessary to select prognostic biomarkers and treatment strategies taking into account the preexisting radiological findings of ILD in the future.

The present study has some limitations. First, the study was limited by the small number of patients and the absence of additional validation datasets. In order to generalize these findings, further validation studies are essential. Second, the clinical diagnoses of the enrolled patients were heterogenous, but there was no significant difference in the ILD diagnoses between the groups with and without CT honeycombing. Third, both groups likely had various pathological changes other than DAD, but pathological assessment was not performed after the onset of AE in all patients due to severe respiratory failure [7]. Therefore, the credibility of this study will be increased by evaluating the relationship between clinical parameters such as blood examinations and radiographic findings and prognosis in autopsy cases only.

## 5. Conclusions

In conclusion, the clinical features of patients with and without honeycombing may differ due to the difference in prognostic factors, but these groups had similar prognoses 3 months after AE onset, and clinicopathological examinations according to these groups are essential.

## Figures and Tables

**Table 1:** Patients' characteristics.

Characteristics	Total patients (*N* = 95)
Age, y	75 (71–80)
Male sex	68 (72)
CCIS	6 (4–7)
From ILDs diagnosis to AE treatment, days	1102 (229–2103)
From symptom onset to treatment, days	6 (3–15)

Diagnosis of AE	
Idiopathic ILDs	
IPF	21 (22)
Non-IPF	41 (43)

Secondary ILDS	
CTD-ILD	19 (20)
Drug-induced ILD	13 (14)
CHP	1 (1)

Physiological parameter	
P/F ratio	268 (188–308)

Biomarkers	
LDH (IU/L)	282 (237–404)
SP-D (ng/mL)	233 (139–407)
KL-6 (U/mL)	897 (563–1553)

Treatment	
PSL before pulse	21 (22)
PSL pulse	95 (100)
PSL after pulse	77 (82)
Macrolide	20 (21)
Anticoagulant	18 (19)

Outcome	
3-month mortality	23 (24)

Results are shown as medians with 25th–75th percentiles or numbers (%). Serum SP-D could be measured in 92 patients (97%). AE, acute exacerbation; CCIS, Charlson Comorbidity Index Score; CTD-ILDs, connective tissue disease-associated ILDs; GGO, ground-glass opacity; HRCT, high-resolution computed tomography; ILD, interstitial lung disease; IPF, idiopathic pulmonary fibrosis; KL-6, Krebs von den Lungen; LDH, lactate dehydrogenase; P/F ratio; partial pressure of oxygen in arterial blood/fraction of the inspiratory oxygen; PSL, prednisolone; SP-D, surfactant protein-D.

**Table 2:** HRCT scores at diagnosis of AE.

Valuables	Survive more than 3 months	Death within 3 months	*P* values
GGO + consolidation score	9.5 (7–13)	12 (9–16)	0.147
Honeycomb score	1 (0–4)	3 (0–8)	0.214
Reticular fibrosis score	1 (0–2)	1 (0–2)	0.531
Honeycomb + reticular fibrosis score	3 (1–5.8)	3 (0–8)	0.782
Total score	14 (9.3–19)	16 (12–23)	0.156
GGO + consolidation score/total score (%)	81.5 (59.3–95.8)	79 (58–100)	1.000
Honeycomb score/total score (%)	5.5 (0–28.5)	17 (0–38)	0.399
Honeycomb + reticular fibrosis score/total score (%)	18.5 (4.3–40.8)	21 (0–42)	1.000

Results are shown as medians with 25th–75th percentiles or percentage (%). These scores were calculated at the diagnosis of AE conditions. AE, acute exacerbation; GGO, ground-glass opacity; HRCT, high-resolution computed tomography.

**Table 3:** Clinical comparison of patients with and without honeycombing.

Characteristics	With honeycombing (*N* = 52)	Without honeycombing (*N* = 43)	Total patients (*N* = 95)	*P* values with vs. without honeycombing
Age, y	78 (73–82)	74 (66–79)	75 (71–80)	0.013
Male sex	35 (67)	33 (76)	68 (72)	0.310
CCIS	6 (5–7)	4.5 (3–8.8)	6 (4–7)	0.411
From ILDs diagnosis to AE treatment, days	1390 (739–2482)	590 (56–1870)	1103 (229–2103)	0.011
From symptom onset to treatment, days	6 (2.8–16.3)	6 (2.3–14)	6 (3–15)	0.128

Physiological parameter				
P/F ratio	266 (202–307)	271 (180–316)	268 (188–308)	0.686

Biomarkers				
LDH (IU/L)	279 (248–340)	290 (210–415)	282 (237–404)	0.173
SP-D (ng/mL)	279 (154–507)	194 (110–366)	233 (139–407)	0.080
KL-6 (U/mL)	1058 (768–1910)	572(310–1202)	897 (563–1553)	0.008

Treatment				
PSL before pulse	12 (23)	9 (21)	21 (22)	0.802
PSL pulse	52 (100)	43 (100)	95 (100)	1.000
PSL after pulse	43 (84)	34 (79)	77 (82)	0.511
Macrolide	6 (12)	14 (33)	20 (21)	0.012
Anticoagulant	9 (17)	9 (21)	18 (19)	0.652

Outcome				
3-month mortality	14 (27)	9 (21)	23 (24)	0.497

**Table 4:** Univariate and multivariate analysis of primary predictors of 3-month mortality.

Variable	Univariates			Multivariates		
	Odds ratio	95% CI	*P*	Odds ratio	95% CI	*P*
*Honeycomb present*						
Age	1.067	0.912–1.087	0.918			
Sex	5.348	0.629–45.479	0.125			
CCIS	1.494	0.970–1.652	0.082			
Diagnosis of ILDs	0.589	0.124–2.797	0.505			
Serum LDH	1.005	1.002–1.008	0.005	1.005	1.002–1.007	<0.001
Serum KL-6	1.000	1.000–1.001	0.454			
P/F ratio	0.998	0.990–1.006	0.580			
GGO score	0.886	0.723–1.085	0.244			

*Honeycomb absent*						
Age	1.066	0.953–1.216	0.270			
Sex	4.174	0.183–95.180	0.340			
CCIS	1.494	1.098–2.311	0.009	1.436	1.156–1.842	0.001
Diagnosis of ILDs	3.389E + 10	0	1.000			
Serum LDH	1.004	0.994–1.015	0.996			
Serum KL-6	1.000	0.999–1.001	0.906			
P/F ratio	1.004	0.982–1.006	0.425			
GGO score	1.060	0.897–1.247	0.482			

CCIS, Charlson Comorbidity Index Score; CI, confident interval; GGO, ground-glass opacity; ILD, interstitial lung disease; LDH, lactate dehydrogenase; KL-6, Krebs von den Lungen; P/F ratio, partial pressure of oxygen in arterial blood/fraction of inspired oxygen.

## Data Availability

The datasets used and/or analyzed during the current study are available from the corresponding author upon request.

## Abbreviation

AE: Acute exacerbation
AUC: Area under the ROC curve
CCIS: Charlson Comorbidity Index Score
CHP: Chronic hypersensitivity pneumonia
CI: Confidence interval
CTD-ILDs: Connective tissue disease-associated ILDs
DAD: Diffuse alveolar damage
DAH: Diffuse alveolar hemorrhage
DIILD: Drug-induced interstitial lung disease
GGO: Ground-glass opacity
HRCT: High-resolution CT
IIPs: Idiopathic interstitial pneumonias
ILD: Interstitial lung disease
IPF: Idiopathic pulmonary fibrosis
KL-6: Krebs von den Lungen
LDH: Lactate dehydrogenase
OP: Organizing pneumonia
OR: Odds ratio
P/F ratio: Partial pressure of oxygen in arterial blood/fraction of inspired oxygen
PSL: Prednisolone
ROC: Receiver operating characteristic
SP-D: Surfactant protein-D
UIP: Usual interstitial pneumonia.

## Supplementary Materials

Supplementary Materials Figure S1. Comparison between patients with high honeycomb and reticular fibrosis score and low. Similar with the honeycomb score, there was no significant difference in 3-month mortality among the groups with low and high fibrosis score including reticular fibrosis and honeycomb score. Figure S2. Comparison of patients with and without honeycombing 6 months after AE. There is no significant difference in 6-month mortality between those with and without honeycombing in patients with AEs of idiopathic ILDs, AEs of secondary ILDs, and overall patients. AE, acute exacerbation; ILD, interstitial lung disease. .
