# Supplementary material for: The Extent of Honeycombing on Computed Tomography Cannot Predict the Treatment Outcome of Patients with Acute Exacerbations of Interstitial Lung Disease
Source: Can Respir J. 2021 Nov 16;2021:7456315. doi: 10.1155/2021/7456315 (PMC8610694; doi:10.1155/2021/7456315)

Figure S2.
Comparison between patients with and without honeycombing 6 months after AE

A. Overall

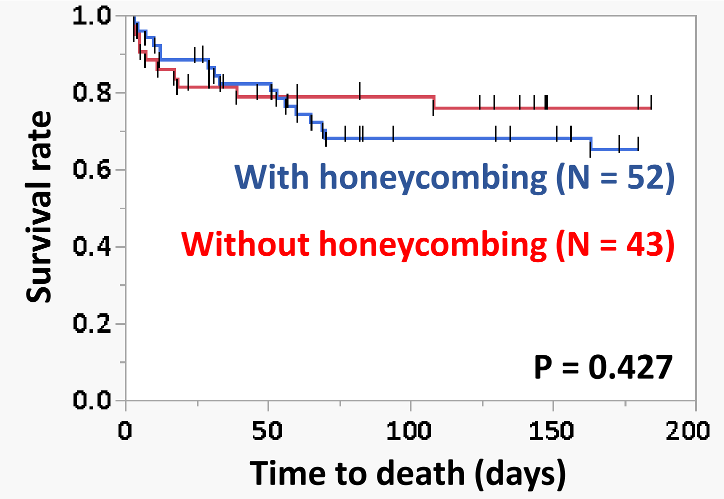

B. Idiopathic

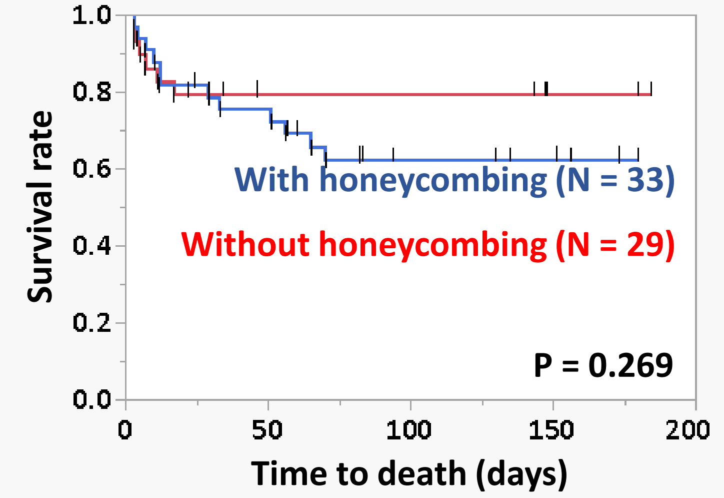

C. Secondary

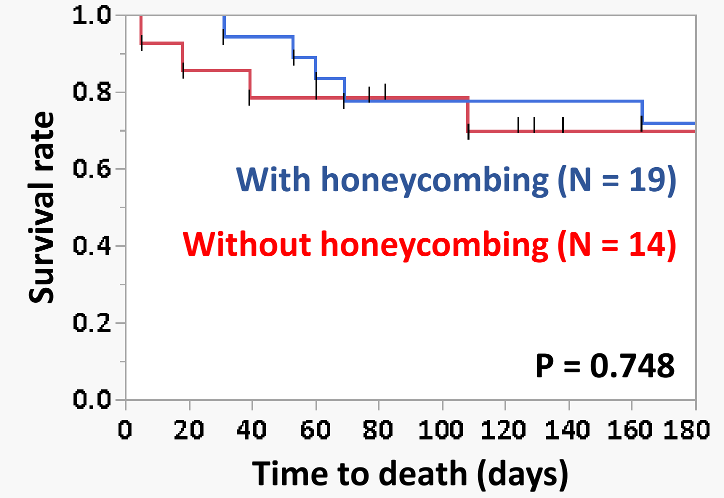

Supplement: Supplementary Materials — Figure S1. Comparison between patients with high honeycomb and reticular fibrosis score and low. Similar with the honeycomb score, there was no significant difference in 3-month mortality among the groups with low and high fibrosis score including reticular fibrosis and honeycomb score. Figure S2. Comparison of patients with and without honeycombing 6 months after AE. There is no significant difference in 6-month mortality between those with and without honeycombing in patients with AEs of idiopathic ILDs, AEs of secondary ILDs, and overall patients. AE, acute exacerbation; ILD, interstitial lung disease. . [file 7456315.f1.zip › 7456315.f1/Figure Supplement 2.pdf]
